# A rare but devastating cause of cardiac tamponade

**DOI:** 10.1007/s12471-019-1287-5

**Published:** 2019-05-09

**Authors:** R. S. Hermanides, A. H. Tavenier, A. P. Nierich

**Affiliations:** 1 Departments of Cardiology, Isala Hospital, Zwolle, The Netherlands; 2 Department of Cardiothoracic Intensive Care and OR, Isala Hospital, Zwolle, The Netherlands

**Fig. 1:**
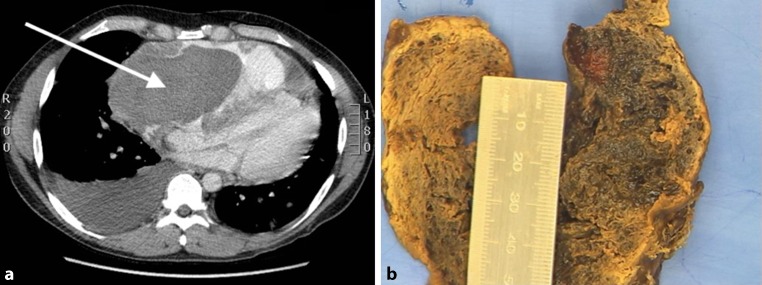
**a** Chest CT image with a very large extra-cardiac mass and 6 cm pericardial effusion with compression of the right atrium and right ventricle. Space-occupying lesion in the right atrium, diffuse lesion in both lungs and in the spleen. Pleural effusion right. **b** Macroscopic image of an angiosarcoma

A 37-year-old male patient was admitted to hospital with a 1-month history of progressive dyspnoea. He had no cardiac history. Cardiovascular examination was highly suspicious for cardiac tamponade. Transthoracic echocardiography and computed tomography (CT) of the chest showed an extra cardiac mass and 6 cm pericardial effusion with compression of the right atrium and ventricle (Fig. 1a). Emergency surgery was performed. There was a haemopericardium due to active bleeding of a fistula between the right coronary artery (RCA) and a large mass (15 × 6 cm) that invaded into the pericardium and epicardium. The mass and a large part of the pericardium were removed and a pericardial patch was placed. As the mass protruded into the coronary artery, revascularisation of the RCA could not be performed. Because of refractory cardiogenic shock due to right ventricular failure the patient could not be weaned from extracorporeal circulation. The patient died on the intensive care unit shortly after surgery. Pathological examination revealed an angiosarcoma (Fig. 1b).

